# Ist das (schon) Hatespeech? – Eine qualitative Untersuchung zum Verständnis von Hatespeech unter pädagogischem Schulpersonal

**DOI:** 10.1007/s35834-022-00367-1

**Published:** 2022-11-23

**Authors:** Cindy Ballaschk, Friederike Schulze-Reichelt, Sebastian Wachs, Norman Krause, Alexander Wettstein, Julia Kansok-Dusche, Ludwig Bilz, Wilfried Schubarth

**Affiliations:** 1 Department Erziehungswissenschaft, Universität Potsdam, Karl-Liebknecht-Str. 24–25, 14476 Potsdam, Deutschland; 2 Institut für Forschung, Entwicklung und Evaluation, Pädagogische Hochschule Bern, Fabrikstrasse 8, 3012 Bern, Schweiz; 3 Fakultät für Soziale Arbeit, Gesundheit und Musik, Brandenburgische Technische Universität Cottbus-Senftenberg, Universitätsplatz 1, 01968 Senftenberg, Deutschland

**Keywords:** Hatespeech, Schule, Pädagog_innen, Hatespeech-Verständnis, Hate Speech, School, Pedagogues, Hatespeech Understanding

## Abstract

Hatespeech ist ein gesellschaftliches Phänomen, das auch die Lebenswelt Schule betrifft. Was jedoch pädagogisches Schulpersonal unter Hatespeech versteht, ist unklar und daher Thema der vorliegenden Interviewstudie mit Lehrkräften (*n* = 18) und Sozialpädagog_innen (*n* = 16). Die Ergebnisse zeigen, dass Hatespeech als Herabsetzung verschiedener strukturell unterdrückter Gruppen verstanden wird (z. B. Transpersonen, Homosexuelle, jüdische Personen). Die Befragten erkennen subtilere Formen von Hatespeech oft nicht als Problem an und verstehen Hatespeech vorranging als Online-Phänomen, obwohl auch Vorfälle in Schulen berichtet werden. Es lässt sich schlussfolgern, dass pädagogisches Schulpersonal für Offline-Hatespeech und subtilere Formen sensibilisiert werden muss, um seine Interventionsbereitschaft zu steigern.

## Abstract (en)

Hate speech is a social phenomenon that also affects the school environment. However, what school staff understands by hate speech is unclear, and therefore, the objective of the present interview study with teachers (*n* = 18) and social pedagogues (*n* = 16). The results showed that hate speech is understood as the degradation of structurally oppressed groups (e.g., trans persons, homosexuals, Jewish people). Participants often did not recognize subtler forms of hate speech as a problem and understood hate speech primarily as an online phenomenon, even when incidents were reported in schools. It can be concluded that educational school staff needs to be sensitized to offline hate speech and subtler forms to increase their willingness to intervene.

## Einleitung

Hatespeech wird in diesem Beitrag als bewusste Herabsetzung verstanden, bei der Personen gruppenbezogen (z. B. als Homosexuelle, People of Color, Transpersonen) angegriffen, gedemütigt oder dehumanisiert werden (Sponholz 2018; Wachs et al. 2021). Dabei ist Hatespeech von angrenzenden Phänomenen wie z. B. Bullying und Diskriminierung abzugrenzen. Während sich Hatespeech gegen soziale Gruppen richtet und auch einmalig auftreten kann, findet Bullying systematisch und wiederholt statt und kann auch ausgrenzendes Verhalten und physische Gewalt beinhalten. Ein Gruppenbezug muss hier nicht vorliegen, kann aber. Diskriminierung bezieht sich wie Hatespeech auf soziale Gruppen, kann sich, im Gegensatz zu Hatespeech, welche absichtlich eingesetzt wird, auch unbeabsichtigt vollziehen (Wachs et al. 2021). Studien zeigen, dass Jugendliche an deutschen Schulen zwar mit Hatespeech konfrontiert sind (Ballaschk et al. 2021; Krause et al. 2021), der Begriff jedoch wenig bekannt ist und unterschiedlich definiert wird (Papcunová et al. 2021). Um Schüler_innen vor dehumanisierenden Adressierungen zu schützen, ist es u. a. die Aufgabe des pädagogischen Schulpersonals, Hatespeech zu erkennen und einzuschreiten. Doch wie ziehen Pädagog_innen die Grenze zwischen tolerierbaren Äußerungen und nicht akzeptierbaren gruppenbezogenen Abwertungen? Die Beantwortung dieser Frage ist relevant, weil die Gewaltforschung gezeigt hat, dass ein breitgefächertes Gewaltverständnis bei Lehrkräften mit einer höheren Wahrscheinlichkeit in Verbindung dazu steht z. B. bei Bullying zu intervenieren, als ein engeres Gewaltverständnis (Bilz et al. 2016). Zum Hatespeech-Verständnis und Interventionsverhalten von pädagogischem Schulpersonal fehlen bislang derartige Forschungsbefunde. Daher untersucht der vorliegende Beitrag, woran Pädagog_innen Hatespeech erkennen. Die Ergebnisse liefern wichtige Hinweise für die Aus- und Fortbildung von Pädagog_innen, um Hatespeech an Schulen konsequent und erfolgreich entgegentreten zu können.

## Forschungsstand

In Anlehnung an Sponholz (2020) ist der folgende Forschungsüberblick zu Hatespeech unter jungen Menschen entsprechend der Betroffenengruppen, der Ausdrucksformen und der Orte von Hatespeech systematisiert. In Bezug auf die Frage, wer von Hatespeech betroffen ist, sahen Begründer_innen der Hatespeech-Forschung ausschließlich „historically oppressed group[s]“ (Matsuda 1989, S. 2357) als potenzielle Adressat_innen von Hatespeech an (Sponholz 2020). Inzwischen existieren auch Ansätze, die weitere soziale Gruppen einbeziehen, die nicht als historisch unterdrückt gelten, z. B. Journalist_innen (Meibauer 2013; Zick und Preuß 2021). In einer aktuellen Studie berichteten junge Erwachsene (18–25 Jahre) aus sechs Ländern (USA und Europa), im Internet am häufigsten rassistische Hatespeech (37 %) wahrgenommen zu haben, gefolgt von Hatespeech gegen Menschen aufgrund ihrer sexuellen Orientierung (34 %), ihrer Herkunft (33 %), ihres Geschlechts (Sexismus, Transfeindlichkeit; 27 %), ihrer Religionszugehörigkeit (25 %), ihrer politischen Überzeugung (24 %), ihres Aussehens (Lookism; 18 %) und ihrer Behinderung (Ableism; 9 %; Reichelmann et al. 2020).

Bei den Ausdrucksformen von Hatespeech verweist die Forschung auf Kommunikationsformen, bei denen betroffene Personen und Personengruppen absichtlich direkt oder indirekt kommunikativ abgewertet werden (May 2018; Meibauer 2013; Wachs et al. 2020). Während in manchen Fällen Hatespeech unmissverständlich als solche erkennbar ist, kann diese auch auf latente und suggestive Weise ausgedrückt werden (Stefanowitsch 2015). Als offensichtlich zeigt sich Hatespeech, wenn sie sich in Form gruppenbezogener verunglimpfender Begriffe, Beleidigungen, Drohungen oder gar konkreten Aufrufen zu Gewalt zeigt. Hatespeech kann aber ebenfalls als Ironie oder Humor getarnt werden sowie sich in Form einer bewussten Verbreitung von Halb- und Unwahrheiten, Generalisierungen und Gleichsetzungen und oft in Form von Stereotypen zeigen (Ballaschk et al. 2021; Krause et al. 2021; Paasch-Colberg et al. 2021; Reichelmann et al. 2020). Insbesondere bei Stereotypen, die harmlos oder positiv wirken, wird Hatespeech seltener erkannt (Stefanowitsch 2015). Der Begriff Hatespeech wird meist mit Hass, also einer starken Abneigung oder einem Gefühl der Ablehnung und Feindschaft gegenüber einer Personengruppe assoziiert. Doch Hatespeech kann auch emotionslos, gezielt und berechnend zur Erreichung instrumenteller Ziele eingesetzt werden (Ballaschk et al. 2021; Sponholz 2018).

Als Ort von Hatespeech wird aktuell vorrangig die digitale Sphäre erforscht (Eickelmann 2017; Zhang et al. 2018). Erste Forschungsarbeiten zu dem Thema betrachteten Hatespeech in der analogen Welt (Matsuda 1989). Heute berichten Jugendliche von Hatespeech-Erfahrungen sowohl offline als auch online. In einer Befragung unter Heranwachsenden in Großbritannien zeigte sich, dass die Befragten Hatespeech am häufigsten im Internet beobachtet haben (82 %), dicht gefolgt von Hatespeech in der Schule (77 %), in den Massenmedien (69 %, z. B. Zeitungen, TV) und anderen Offline-Lebenswelten (z. B. 54 % Familie; UK Safer Internet Centre 2016).

Die Skizzierung des Forschungsstands verdeutlicht die große Vielfalt von Hatespeech hinsichtlich der Betroffenen, der Ausdrucksformen und der Erscheinungsorte. Diese unterschiedlichen Charakteristiken können das Eingrenzen und Abgrenzen von Hatespeech zur Herausforderung werden lassen. Dabei stellt sich auch die Frage: Wie können Schulen Hatespeech begegnen, wenn unklar ist, woran pädagogisches Schulpersonal (z. B. Lehrkräfte, Schulsozialarbeiter_innen) Hatespeech festmacht? Verschiedene Studien aus dem Bereich der Diskriminierungsforschung zeigten, dass Pädagog_innen auf diskriminierende Beleidigungen teils mit Abwehr, Relativierungen und Bagatellisierungen reagieren, weil sie dies als normalen Bestandteil jugendlicher Lebenswelten betrachten (Bernstein und Diddens 2021; Chernivsky und Lorenz 2020; Klocke et al. 2018). Aus der Gewaltforschung ist bekannt, dass Lehrkräfte eher eingreifen, wenn sie ein breiteres Gewaltverständnis haben, das z. B. auch soziale Ausgrenzung einschließt (Bilz et al. 2016). Vor diesem Hintergrund erscheint es notwendig zu untersuchen, woran pädagogisches Schulpersonal Hatespeech festmacht und wo Grenzen zu legitimen Meinungsäußerungen gezogen werden. Hier setzt der vorliegende Beitrag anhand der folgenden drei Leitfragen an:

1. Wer ist aus Sicht des pädagogischen Schulpersonals von Hatespeech betroffen?
2. Welche Ausdrucksformen von Hatespeech schildern befragte Pädagog_innen?
3. Wo nimmt das befragte Schulpersonal Hatespeech wahr?

## Methodisches Vorgehen

### Sampling und Feldzugang

Die Stichprobe basiert auf Interviews mit 18 Lehrkräften und 16 Schulsozialarbeiter_innen unterschiedlicher Geschlechter. In Berlin wurden 21 und in Brandenburg 13 Personen befragt. An der Studie nahmen fünf Schulen in Berlin und vier in Brandenburg teil. Aufgrund der Schulschließungen durch die Covid-19-Pandemie konnte an zwei Schulen in Brandenburg nur je eine Lehrkraft befragt werden. Im Sinne eines „purposeful sampling“ (Palinkas et al. 2015) fand vorab eine kriteriengeleitete und möglichst kontrastreiche Fallauswahl statt. In Berlin wurden Schulen aus sozioökonomisch stärkeren mit schwächeren Stadtteilen sowie nach Ost- und Westberlin kontrastiert. In Brandenburg wurden Schulen in städtischen und ländlichen Gebieten befragt. Im Sample befanden sich vier Gymnasien, zwei Oberschulen und drei integrierte Sekundarschulen. Verschiedene Netzwerke, bildungspolitische Akteur_innen, weitere Gatekeeper sowie direkte Anfragen ermöglichten den Zugang zum Forschungsfeld.

### Interviews

In episodischen Interviews (Flick 2011) wurden mit Hilfe eines Interviewleitfadens offene Erzählaufforderungen zu berufsbiografischem, erfahrungsnahem und situationsbezogenem Wissen formuliert und durch konkrete, zielgerichtete Fragen zu semantischem Wissen ergänzt. Um im Sinne einer maximalen Offenheit den Befragten eine möglichst unbeeinflusste Darstellung ihrer subjektiven Sichtweisen zu ermöglichen, wurde eine sehr offene Erzählaufforderung gewählt, um herauszufinden, welche Konzeptionen von Hatespeech Befragte haben: „Erzählen Sie mir zunächst bitte, was Sie mit dem Begriff Hatespeech verbinden“. Erst danach wurde die Arbeitsdefinition zu Hatespeech mit Hilfe eines Schaubildes wie folgt erläutert:

„Hatespeech heißt auf Deutsch Hassrede. Wir verstehen darunter, dass eine Personengruppe öffentlich, absichtlich und abwertend beleidigt wird. Manchmal wird auch eine einzelne Person beleidigt, weil sie zu einer bestimmten Gruppe gehört, beispielsweise aufgrund der Religion (z. B. muslimisch, jüdisch), einer Behinderung, der sexuellen Orientierung (z. B. lesbisch, schwul), der Hautfarbe (z. B. Menschen mit dunklerer Hautfarbe), des Geschlechts (z. B. Mädchen, Transpersonen) oder des Aussehens (z. B. dick sein). Diese Beleidigung kann hier an der Schule passieren. Sie kann aber auch online stattfinden, z. B. über WhatsApp-Klassenchats, Snapchat oder Twitter.“

Diese Arbeitsdefinition inklusive der beispielhaften Nennung sozialer Gruppen hat sich in den Pretests als notwendig erwiesen, da den Befragten der Gruppenbezug von Hatespeech nicht deutlich genug war und die Abgrenzung zu Bullying schwerfiel. Die Erläuterung diente der Absicherung, dass Befragte sich in ihren weiteren Ausführungen auf Hatespeech und nicht auf verwandte Phänomene wie Bullying oder auf Beleidigungen ohne Gruppenbezug beziehen.

### Analysestrategien

Die Interviews wurden in Anlehnung an das Transkriptionssystem GAT2 (Dresing und Pehl 2015) softwaregestützt transkribiert. Entsprechend der Fragen des Interviewleitfadens wurden deduktiv fünf Oberkategorien festgelegt: ‚Definitionen von Hatespeech‘, ‚Gruppenbezüge von Hatespeech‘ (z. B. rassistische Hatespeech), ‚Formen von Hatespeech‘ (z. B. Stereotype), ‚Motive und Ursachen‘ sowie ‚Umgang und Folgen von Hatespeech‘. In Anlehnung an die Grounded Theory (Strauss und Corbin 2010) wurde im Zuge des Auswertungsprozesses innerhalb dieses deduktiv festgelegten Kategoriensystems induktiv codiert, teils in vivo. Beispielsweise wurden innerhalb der Kategorie ‚Definitionen von Hatespeech‘ die Codes ‚Verleumdungen‘, ‚Stereotypen‘, ‚humoristische Tarnung‘ und der ‚Zusammenhang mit Gewalt und Bullying‘ vergeben. Im Rahmen der Analyse wurden Kategorien zusammengefasst, wenn sich starke Überschneidungen zeigten. Andere Codes wurden voneinander abgegrenzt und modifiziert (axiales und selektives Codieren). Dieser Prozess fand softwaregestützt mit MAXQDA statt. Ziel war es, codierte Textstellen der Kategorie ‚Definitionen von Hatespeech‘ kontrastiv zu interpretieren, um einen übergeordneten sozialen Sinn zu rekonstruieren (Ullrich 2020). Dadurch sollen Wissens- und Bedeutungsstrukturen der Befragten zu ihrem Verständnis von Hatespeech freigelegt werden. Dies geschieht, indem codierte Textstellen immer wieder auf ihre Ähnlichkeiten und Unterschiede geprüft werden (Strübing 2014). Als Vergleichskriterien der kontrastiven Analyse wurde die Struktur der drei Forschungsfragen zugrunde gelegt (Betroffene, Ausdrucksformen und Orte von Hatespeech). Um Unterschiede in den Perspektiven auf Hatespeech sichtbar zu machen und somit die Bandbreite des Gegenstandsbereiches darzustellen, wurde die Vergleichsstrategie des maximalen Vergleichs gewählt (Ullrich 2020). Das bedeutet, dass möglichst unterschiedliche Textstellen miteinander in Beziehung gesetzt wurden.

## Ergebnisse

### Wer ist aus Sicht des pädagogischen Schulpersonals von Hatespeech betroffen?

Die Interviewten verstehen Hatespeech überwiegend als eine abwertende Kommunikation, die sich gegen Angehörige bestimmter Gruppen richtet, z. B. als „menschengruppenbezogene Beleidigung“ (L[ehrkraft]04). Dabei werden verschiedene soziale Gruppen als betroffen von Hatespeech an der Schule genannt. Vorrangig beziehen sich die Interviewten auf strukturell unterdrückte Gruppen. Die Befragten schildern häufig, rassistische Hatespeech und Hatespeech gegen bisexuelle, homosexuelle und transgeschlechtliche Personen wahrzunehmen. Auch Hatespeech gegen Mädchen und Frauen sowie gegen Personen muslimischen und jüdischen Glaubens werden oft genannt. Vergleichsweise selten wird Hatespeech gegen Menschen mit Behinderung oder einer bestimmten sozialen Herkunft berichtet. Teils werden auch Herabwürdigungen sozialer Gruppen erwähnt, die nicht als strukturell unterdrückt gelten. Dabei handelt es sich meist um Schüler_innen, die sich in irgendeiner Form von ihren Mitschüler_innen abheben, z. B. Vegetarier_innen. Es fällt auf, dass Pädagog_innen unterschiedliche Perspektiven bei Verunglimpfungen von sozialen Gruppen einnehmen. Dies wird im Weiteren exemplarisch anhand von Berichten über transfeindliche und antisemitische Herabsetzungen dargestellt. Transfeindlichkeit und Antisemitismus wurden ausgewählt, weil sich hier am Interviewmaterial das Hatespeech-Verständnis und Abwägungsprozesse der Befragten besonders anschaulich kontrastieren lassen.

#### Transfeindliche Äußerungen

Pädagog_innen schildern unterschiedliche Degradierungen gegenüber transgeschlechtlichen Schüler_innen und Lehrkräften. Folgende Lehrkraft sieht in den dargestellten Herabsetzungen keine Anzeichen für transfeindliche Hatespeech:

Beispiel 1: „Ja, wir hatten schon mal welche, die gerne Männlein statt Weiblein geworden sind. Ja, das wird dann so ein bisschen verlacht, aber als Hass würde ich das, so Hassrede, nein. Das wird so ein bisschen verlacht, aber sowas ist dann mal zwei, drei Tage und dann ist wieder, nein, das spielt keine große Rolle“ (L07).

Eine andere Lehrkraft berichtet Herabwürdigungen von Schüler_innen gegenüber einem transgeschlechtlichen Kollegen. Dies wird als transfeindliche Hatespeech eingeordnet:

Beispiel 2: „[D]ie Geschichte wird Ihnen schon mitgeteilt worden sein, welche dann auch durch die Presse ging bis hoch zum Verwaltungsgericht, wo Schülerinnen aus dieser Schule suspendiert wurden. Da ging es um die üble Nachrede gegenüber einem Kollegen, der transsexuell ist. […] Und ich bin auch mit der betroffenen Person eng befreundet und wir stehen auch im regelmäßigen Austausch und von daher, alles was in diese Richtung Hatespeech [geht] […], bin ich einer der ersten, der auch da auf die Barrikaden geht“ (L12).

Beide Beispiele zeigen, dass Herabsetzungen gegen transgeschlechtliche Personen sehr unterschiedlich eingeordnet werden können. Ob diese als Hatespeech bewertet werden, hängt demnach neben der Zugehörigkeit zu einer sozialen Gruppe von weiteren Kriterien ab. In Beispiel 1 wird ein Verlachen transgeschlechtlicher Schüler_innen berichtet, dass nicht als Hatespeech angesehen wird, da keine Anzeichen für Hass festgestellt werden. Mit der Formulierung „die gerne Männlein statt Weiblein geworden sind“, deutet sich eine tendenziell gleichgültige Haltung gegenüber transgeschlechtlichen Lebensweisen an. Beschriebene Degradierungen gegenüber transgeschlechtlichen Schüler_innen werden als humoristisch klassifiziert: „Das wird so ein bisschen verlacht“. Zudem werden die Begebenheiten auf „zwei, drei Tage“ begrenzt gesehen, weswegen ihnen keine große Bedeutung beigemessen wird: „das spielt keine große Rolle“. Hinter der Abwägung des Auslachens als Hass, könnte eine Prüfung der Beweggründe der Ausübenden stehen. Die Perspektive der Betroffenen wird nicht thematisiert. In Beispiel 2 wird die üble Nachrede gegen einen transgeschlechtlichen Lehrer geschildert und dieser damit als betroffen von Hatespeech betrachtet. Es wird eine Begebenheit berichtet, die sich gegen eine befreundete Lehrkraft richtete. Verstärkt wird dies durch den Verweis auf fortlaufende Diskriminierungsereignisse, die als strafrechtlich relevant hervorgehoben werden: „[das] ging hoch bis zum Verwaltungsgericht“. Es werden Sanktionen gegen Schüler_innen genannt: „wo Schülerinnen aus dieser Schule suspendiert wurden“. Die Motivation der Schüler_innen wird in dieser Erzählung nicht geprüft. Vielmehr wird auf den rechtswirksamen Tatbestand der „üble[n] Nachrede“ verwiesen und damit eher aus der Perspektive des betroffenen Lehrers argumentiert. Die Sicht auf transgeschlechtliche Personen als potenzielle Adressat_innen von Hatespeech zeigt sich damit nicht nur als vielfältig, sondern auch als abhängig von der Fokussierung auf Ausübende oder auf Betroffene von Herabsetzungen (einschließlich der Beziehung zwischen Betroffenen/Beobachtenden sowie der Bewertung der Geschehnisse z. B. als Hass oder als Straftat).

#### Antisemitische Äußerungen

Das interviewte pädagogische Personal berichtet von verschiedenen abwertenden Äußerungen gegenüber Personen muslimischen und jüdischen Glaubens. Manche Befragte sehen darin Hatespeech und andere nicht:

Beispiel 3: „[A]lle Muslime sind Terroristen bis hin alle Juden sind, was weiß ich […]. Also manchmal hört man so Aussagen, aber die würde ich jetzt nicht als wirkliche Parole oder so als komplett als Hatespeech […] Meistens ist es ja eine Art Provokation, um zu gucken, wie dein Gegenüber […] reagiert […] Und das zähle ich nicht gleich unter Hatespeech, weil ich denke, die Menschen, die halt wirklich diese Hassreden ausführen, dass die sich auch gar nicht von ihrer Meinung überzeugen lassen von anderen“ (S[chulsozialarbeiter_in]03).

Eine andere pädagogische Fachkraft berichtet von Äußerungen eines Schülers über jüdische Personen, die als Hatespeech gewertet werden:

Beispiel 4: „Wo [ein Schüler] relativ unvermittelt davon gesprochen hat, er hasse alle Juden, und die sollten alle sterben. Da war […] kein jüdischer Jugendlicher dabei. Aber für mich ist das trotzdem Hatespeech“ (S05).

Beide Sozialarbeiter_innen beziehen sich auf negative Äußerungen gegenüber Personen jüdischen Glaubens und positionieren sich unterschiedlich in Bezug auf Hatespeech. In Beispiel 3 wird der Inhalt der Äußerungen einer Intention bzw. den Einstellungen von Schüler_innen gegenübergestellt. Die Aussagen werden als „Provokation“ gedeutet. Manifestierte antisemitische Einstellungen werden bei den Schüler_innen nicht gesehen und daher wird keine Hatespeech gegen jüdische Personen festgestellt. Argumentiert wird ebenso wie in Beispiel 1 aus der Perspektive der ausübenden Schüler_innen, hier in Form ihrer Motivation und Einstellungen. Die Auswirkungen auf Adressierte werden nicht thematisiert. In Beispiel 4 wird wie in Beispiel 2 der Inhalt der Wortmeldung des Schülers mit der Möglichkeit potenziell anwesender Betroffener, also jüdischer Schüler_innen im Raum, in Beziehung gesetzt. Die Artikulation von Hass und Todeswünschen wird als derart schwerwiegend betrachtet, dass trotz der Abwesenheit jüdischer Personen Hatespeech festgestellt wird. Dies verweist auf ein Verständnis von Hatespeech, welches sich auch in Abwesenheit der adressierten Gruppe vollziehen kann. In diesem Beispiel wird somit einerseits Hass konstatiert und andererseits die Perspektive der Adressierten einbezogen, die möglicherweise zugegen sein könnten. An den Zitaten wird deutlich, dass die Bewertung der Schwere der Äußerungen mit der Motivation der Ausübenden bzw. der Auswirkungen auf Betroffene für oder gegen eine Einordnung als Hatespeech zusammengebracht wird.

Die vier Beispiele machen deutlich, dass die Einordnung einer sozialen Gruppe als von Hatespeech betroffen, von der Beurteilung der Schwere einer Aussage und der Fokussierung auf Ausübende oder Betroffene von Hatespeech abhängt. Diese Tendenz wird im gesamten Interviewmaterial sichtbar. Somit lässt sich für die erste Forschungsfrage festhalten, dass soziale Gruppen eher als vulnerabel angesehen werden, wenn die Perspektive der Adressierten eingenommen wird und das Geschehene als schwerwiegend, als Hass oder Feindseligkeit gewertet wird. Daraus lässt sich vermuten, dass bei der Beurteilung von Hatespeech weniger die soziale Gruppe von Bedeutung ist, sondern stärker die Art und Weise sowie der Kontext relevant erscheinen, in dem Hatespeech auftritt. Dies führt zur Forschungsfrage nach den Ausdrucksformen von Hatespeech.

### Welche Ausdrucksformen von Hatespeech schildern befragte Pädagog_innen?

Interviewte nehmen verschiedene Formen von Hatespeech wahr. Demnach adressieren sich Schüler_innen gegenseitig teils sehr direkt, z. B. mit Verunglimpfungen und Beleidigungen wie: „Du bist hässlich, weil du schwarz bist“ (L11). Hatespeech wird auch als indirekte Form im Schulalltag berichtet, z. B., wenn Schüler_innen hinter dem Rücken übereinander reden und sich an der „Verbreitung von Halb- oder Unwahrheiten“ (L12) beteiligen. Zudem werden Generalisierungen und Gleichsetzungen bis hin zu Drohungen und Aufrufen zu Gewalt benannt. Innerhalb dieser mehr oder weniger direkten Ausdrucksformen lässt sich die geschilderte Hatespeech noch weiter ausdifferenzieren, und zwar in Verleumdungen, Sarkasmus, Stereotypen, humoristischer Tarnung und abwertende Sprachriten, Aufforderung zur Selbstverletzung sowie in Zusammenhang mit physischer Gewalt und Bullying. In den Schilderungen der Befragten zeigen sich dabei Überschneidungen, z. B. sind stereotype und sarkastische Äußerungen oft humoristisch getarnt. Aus dem Interviewmaterial wird sichtbar, dass je drastischer die Ausdrucksform von Hatespeech empfunden wird, desto eher wird Hatespeech konstatiert. So sind sich die meisten Pädagog_innen einig, dass Gewaltandrohungen als Hatespeech gelten. Uneinigkeit besteht hingegen bei subtileren Formen von Hatespeech, z. B. bei humoristisch getarnten Herabsetzungen. Welche Aspekte bei verschiedenen Ausdrucksformen eine Bewertung für oder gegen Hatespeech beeinflussen, wird exemplarisch am Beispiel von Stereotypen sowie von humoristisch getarnter Hatespeech und abwertender Sprachriten dargestellt.

#### Stereotype

Pädagog_innen berichten aus ihrem Schulalltag von stereotypen Aussagen und gruppenbezogenen Unterstellungen. Diese werden, wie in Beispiel 5, nicht immer als Hatespeech betrachtet. Eine Lehrkraft benennt „rassistische Vorurteile“ (L08) von Seiten des Kollegiums gegenüber Schülern: „Indem z. B. gesagt wird: ‚Der wird ja gut tanzen können, der und der Junge‘“ (L08). Diese pauschalisierenden Aussagen werden von dieser Lehrkraft wie folgt eingeordnet:

Beispiel 5: „Hatespeech [ist es] eben nicht, sondern [die] Reproduktion von Vorurteilen. […] nicht als Schimpfwort, sondern als einfach offene Äußerung von Vorurteilen.“ (L08).

Während hier Stereotype nicht als Hatespeech betrachtet werden, schildert eine andere Lehrkraft ebenfalls die Verbreitung von Vorurteilen, die als Hetze angesehen werden:

Beispiel 6: „Da würde ich fast sagen, je mehr Bildung, desto mehr wird gegen Hartz IV gehetzt. So habe ich den Eindruck bei den Schülerinnen und Schülern. Das ist sehr extrem. ‚Die liegen nur rum. Die sind sowieso faul. Die wollen nicht arbeiten‘.“ (L02)

An den Zitaten zeigt sich, dass stereotype Herabsetzungen nicht immer als Hatespeech bewertet werden. So lehnt es die Lehrkraft in Beispiel 5 ab, als rassistisch benannte Vorannahmen von Kolleg_innen als Hatespeech einzuordnen. Es wird betont, dass es sich dabei um eine „Reproduktion“ oder „offene Äußerung von Vorurteilen“ handelt. Mit der Formulierung „nicht als Schimpfwort“ deutet sich eine Prüfung der Verletzungsabsicht der äußernden Kolleg_innen an, die nicht bestätigt wird. Damit wird wie im Beispiel 1 und 3 aus der Perspektive der Ausübenden und nicht des betroffenen Schülers argumentiert und eine Kategorisierung als Hatespeech abgelehnt. In Beispiel 6 wird nicht explizit von Hatespeech gesprochen, sondern davon, dass unter Schüler_innen mit höherem Bildungsniveau viel über Beziehende von Arbeitslosengeld II „gehetzt“ werde. Da Hass und Hetze oft im Zusammenhang von Hatespeech benannt werden, wird eine Nähe zur Bewertung als Hatespeech hergestellt. Auch hier werden ausschließlich Aspekte der Ausübenden und nicht der Betroffenen reflektiert, wenn auf den Bildungshintergrund der äußernden Schüler_innen eingegangen wird. An den Zitaten wird deutlich, dass die Bewertung der Äußerungen als Vorurteil bzw. als Hetze mit einer Beleidigungsabsicht der Kolleg_innen bzw. der sozialen Herkunft der Schüler_innen verknüpft wird und darüber eine Klassifikation der Äußerungen stattfindet.

#### Humoristische Tarnung und abwertende Sprachriten

Befragte berichten von Hatespeech, die sich teilweise „als Humor getarnt“ (S07) zeigt. Das bedeutet, dass z. B. teils verfassungsfeindliche Inhalte in Memes und Stickern verschickt werden, die als lustig wahrgenommen werden:

„Da gab es über irgendeine WhatsApp-Gruppe […] irgendein Bild. […] Wo eine Wolke zu sehen war und in der Wolke stand drinnen: ‚Familienbild einer jüdischen Familie‘. Und […] jeder Schüler hat sich da dran quasi hochgegeilt“ (L05).

Auch werden diskriminierende Beleidigungen unter Schüler_innen geschildert, die auf Rückfrage oft als Spaß gerechtfertigt werden. Bestimmte diskriminierende Bezeichnungen, wie z. B. „Schwuchtel“ (L11), „Schlampe“ (L13) oder „Jude“ (L02), werden als fest institutionalisierte Sprachriten unter Schüler_innen und als inflationär benutzt dargestellt. Einige Pädagog_innen beschreiben, dass es bei herabsetzenden Schimpfworten „so schwer [sei] auseinander zu halten, was Spaß ist und was nicht“ (S05) und deuten daher spaßintendierte Herabsetzungen teils nicht als Hatespeech:

Beispiel 7: „Es kommt ja auch darauf an, wenn sich da zwei Kumpels gegenseitig öffentlich beleidigen, dann ist das für mich halt kein Hatespeech, wenn das für die beiden okay ist […] also, es ist klar abwertend in der Aussage, aber nicht so gemeint, oder?“ (S05).

Es zeigen sich jedoch auch andere Perspektiven auf den Umgang unter Schüler_innen, wenn darauf verwiesen wird: „hier ist eine Menschengruppe verletzt worden und […] die sagen das ständig, das ist nur als Scherz gemeint“ (L04). Die folgende Lehrkraft betont zudem die Auswirkungen einer solchen Sprachkultur:

Beispiel 8: „Naja, also, wenn z. B. Schwuchtel ein Standard-Schimpfwort ist, dann ist das für mich auch, wenn sozusagen der Beleidigte oder die Beleidigte nicht homosexuell ist, aber trotzdem als Schwuchtel bezeichnet wird, dann finde ich ist das auch Hatespeech, weil sozusagen eine Normalisierung der Erniedrigung aufgrund von sexueller Identität damit stattfindet“ (L08).

An den Zitaten wird deutlich, dass manche abwertende Sprachriten nicht als Hatespeech gedeutet und diese teils akzeptiert werden, wenn sie als Spaß unter Freund_innen angesehen werden. So wird in Beispiel 7 ebenso wie in den Beispielen 1, 3 und 5 die Perspektive der ausübenden Schüler_innen eingenommen, indem über ihr freundschaftliches Verhältnis ihre Intention, zu verletzen, geprüft wird. Es wird zwar eine Abwertung gesehen, die aber durch die Formulierung „nicht so gemeint“ als weniger ernsthaft und damit nicht als Hatespeech eingestuft wird. Beispiel 8 verweist auf die Folgen des beleidigenden Umgangs untereinander. So wird betont, dass Herabsetzungen sozialer Gruppen nicht als Spaß verharmlost werden dürfen und eine Schulkultur kritisiert, welche die Degradierung homosexuellen Begehrens duldet und damit normalisiert. Somit wird die Deutung der Schüler_innen als Spaß explizit abgelehnt und auf die Folgen für potenziell Betroffene und auf Umgangsnormen in der Schule verwiesen. Daraus ableitend findet eine Einordnung als Hatespeech statt. Ob der degradierende Umgang unter Schüler_innen als Hatespeech betrachtet wird, ist davon abhängig, ob Pädagog_innen der Perspektive der Schüler_innen folgen, die dies als Spaß ansehen, oder ob die Folgen für Adressierte fokussiert werden, deren Identität als erniedrigt konstatiert wird.

Wie sich bereits in der Beantwortung der ersten Forschungsfrage angedeutet hat, zeigt sich auch bei der zweiten Forschungsfrage nach den wahrgenommenen Ausdrucksformen von Hatespeech, dass eine Einordnung als Hatespeech von weiteren Abwägungen abhängig ist. Einerseits scheint es relevant zu sein, inwieweit die Perspektive der Ausübenden oder der (potenziell) Betroffenen geprüft wird. Anderseits werden Unterschiede sichtbar, je nachdem, ob das Geschehene als Spaß, als schwerwiegend oder gar als Hass wahrgenommen wird.

### Wo wird Hatespeech wahrgenommen?

Hatespeech wird von den Interviewten vorrangig in der digitalen Welt verortet: „Hatespeech ist für mich vom Begriff her erstmal so ein Ding, was online läuft […] Wo die Schüler sich halt bewegen, ob es dann auf Instagram, WhatsApp oder sonst wo ist“ (S05). Berichtet wird von Hatespeech z. B. in Klassenchats via Bilder, Memes oder Sticker, von Fake Accounts, diskreditierenden Bildern oder Videos, die in sozialen Medien hochgeladen und herablassend kommentiert werden. Sowohl Lehrkräfte als auch Schulsozialarbeiter_innen äußern, Hatespeech online oft nicht zu bemerken: „Teilweise bekommt man es ja leider als Lehrperson nicht wirklich mit. Man kriegt dann nur im Nachhinein irgendwie Screenshots oder Ausschnitte von Chatverläufen“ (L12). Es wird geschildert, dass pädagogisches Personal meist erst dann einbezogen wird, wenn sich die digitale Hatespeech unter Schüler_innen zuspitzt: „Das eskaliert manchmal, und dann kriegen wir es mit“ (L08).

Pädagog_innen berichten aber auch von Orten in der Schule, an denen sie Hatespeech wahrnehmen. Eher selten findet ihrer Ansicht nach Hatespeech im Klassenraum statt, sondern häufiger auf dem Schulflur, dem Schulhof, den Toiletten oder außerhalb der Schule. Schüler_innen versuchen sich so der Kontrolle des pädagogischen Personals zu entziehen: „Die Dinge, die meistens passieren, passieren dann entweder nach der Schule, oder halt irgendwo aufm Schulhof, wo wir nicht da sind“ (S09). Zugleich beschreiben Befragte, dass Hatespeech unter Schüler_innen online und in der Schule häufig nicht zu trennen sei. So wird Hatespeech, die in der Schule stattfindet, digital fortgeführt und umgekehrt: „Das ist eigentlich das Schlimmste, dass diese Beleidigungen im Chat ganz große Auswirkungen im Schulalltag haben“ (L09). Die Forschungsfrage, wo Hatespeech wahrgenommen wird, lässt sich daher zusammenfassend so beantworten, dass Pädagog_innen sie offline in der Schule und online z. B. in Klassenchats registrieren. Zugleich sind sie sich bewusst, dass Schüler_innen Hatespeech so einsetzen, dass sie sie nicht mitbekommen und sie meist nur von drastischen Ausmaßen erfahren.

## Diskussion

Ziel dieses Beitrages war es, auf der Basis einer qualitativen Interviewstudie das Verständnis von Hatespeech unter pädagogischem Schulpersonal anhand der drei Fragen zu beleuchten: 1. Wer ist aus Sicht des pädagogischen Schulpersonals von Hatespeech betroffen? 2. Welche Ausdrucksformen von Hatespeech schildern befragte Pädagog_innen? und 3. Wo nimmt das befragte Schulpersonal Hatespeech wahr? Da sich bei den ersten beiden Forschungsfragen zwei zentrale Muster zeigen, werden diese im Weiteren gemeinsam dargestellt.

### Betroffene und Ausdrucksformen von Hatespeech aus der Sicht pädagogischen Schulpersonals

In Bezug auf die erste Forschungsfrage wurden vorrangig strukturell unterdrückte Gruppen als betroffen von Hatespeech angesehen (z. B. jüdische und homosexuelle Jugendliche sowie ethnisierte und migrantisierte junge Menschen), aber auch weitere soziale Gruppen wurden vereinzelt genannt (z. B. Vegetarier_innen). Abgesehen von Hatespeech aufgrund der politischen Überzeugung, was nicht erwähnt wurde, entsprechen die geschilderten sozialen Gruppen denen der Forschungsliteratur (Hawdon et al. 2015; Reichelmann 2020). Anhand von transfeindlichen und antisemitischen Äußerungen wurde exemplarisch gezeigt, wie unterschiedlich Pädagog_innen mögliche Betroffenengruppen von Hatespeech verhandeln. Das Auslachen transgeschlechtlicher Personen wurde nicht als Hatespeech angesehen, strafrechtlich verfolgte transfeindliche Verleumdungen hingegen schon. Auch antisemitische Äußerungen, die als Provokation angesehen wurden, fanden keine Einordnung als Hatespeech, jedoch die Artikulation von Hass und Todeswünschen. Studien zu Antisemitismus in der Schule bestätigen, dass Lehrkräfte dazu neigen, Diskriminierungen zu trivialisieren, zu entschuldigen und zu ignorieren, wenn sie dahinter keine hasserfüllten oder böswilligen Absichten ihrer Schüler_innen wahrnehmen (Bernstein und Diddens 2021; Chernivsky und Lorenz 2020). Auch Studien zu LGBTIQ-Jugendlichen unterstreichen die Tendenz von Pädagog_innen queerfeindliche Vorkommnisse zu bagatellisieren (Klocke et al. 2018; Klocke 2020).

Zur Forschungsfrage nach den Ausdrucksformen von Hatespeech schilderten Pädagog_innen Hatespeech, die sie sehr direkt und klar erkennbar wahrnahmen (z. B. im Zusammenhang mit physischer Gewalt und Bullying) bis hin zu indirekten und subtileren Formen (z. B. Verleumdungen und Sarkasmus). Dies entspricht den bekannten Ausdrucksformen von Hatespeech in der Forschungsliteratur (Paasch-Colberg et al. 2021; Reichelmann et al. 2020). Sehr offene Degradierungen klassifizierten die Befragten in der Regel als Hatespeech, während sie bei eher verdeckten Herabsetzungen unterschiedlich reagierten. Am Beispiel von Stereotypen sowie von humoristischer Tarnung und abwertenden Sprachriten wurde gezeigt, dass diese nicht immer als Hatespeech eingeordnet wurden. Stereotype wurden in einem Beispiel als Hetze wahrgenommen und in einem anderen nicht als Hatespeech angesehen, wenn keine Beleidigungsabsicht dahinter erkannt wurde. Humoristisch gedeutete Degradierungen und abwertende Sprachriten wurden als Hatespeech betrachtet, wenn eine Normalisierung der Erniedrigung betont wurde, jedoch nicht, wenn sie als einvernehmlicher Spaß unter Freund_innen gedeutet wurden. Dass sowohl stereotype Aussagen als auch spaßig intendierte Beleidigungen tendenziell seltener als Hatespeech wahrgenommen werden, bekräftigen linguistische Studien zu Hatespeech (Technau 2017, 2021). Da Hatespeech jedoch häufig in Form von stereotypen Äußerungen dargeboten wird (Paasch-Colberg et al. 2021; Reichelmann et al. 2020), müssen Pädagog_innen auch subtilere Formen von Hatespeech erkennen, denn Stereotype fördern die Spaltung sozialer Gruppen (Hall 1997). Es ist wichtig Stereotype ernst zu nehmen, weil Stereotype die Grundlage für menschenfeindliche Ideologien darstellen (Thiele 2019). Darüber hinaus ist aus der Forschung zu Online-Hatespeech bekannt, dass vorrangig wenig ideologisch überzeugte Personen Hatespeech spaßmotiviert einsetzen (Schmitt 2017). Daher ist es notwendig, dass Pädagog_innen die diskriminierende Wirkung auch bei humoristisch gedeuteten Herabsetzungen erkennen und eingreifen.

**Figure 1:**
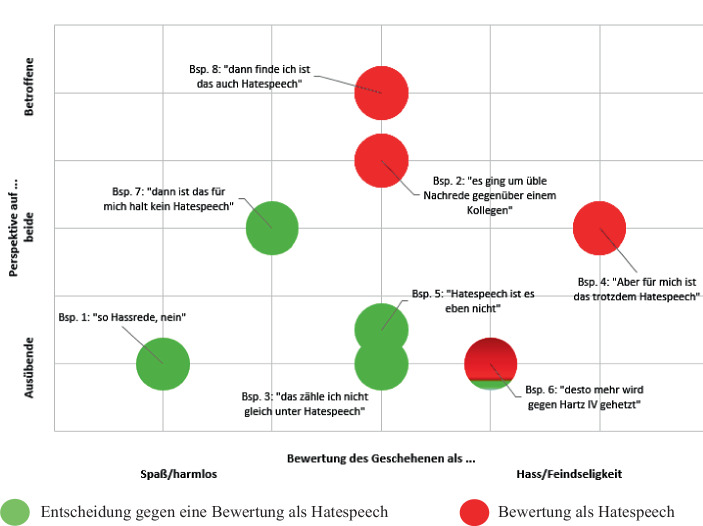

Für die ersten beiden Forschungsfragen, wer von Pädagog_innen als betroffen von Hatespeech angesehen wird und welche Ausdrucksformen wahrgenommen werden, zeigen sich im Interviewmaterial zwei zentrale Muster. Wie in Abb. 1 dargestellt, bewerten Befragte das Geschehene tendenziell eher als Hatespeech, wenn sie die Perspektive der von Hatespeech Adressierten einnehmen und weniger die der Ausübenden von Hatespeech. Zudem erkennen sie eher Hatespeech, wenn sie Aussagen als schwerwiegend oder gar als hasserfüllt bzw. feindselig bewerten und weniger als Spaß oder Provokation deuten.

Dass Diffamierungen eher als Hatespeech angesehen werden, je mehr die Perspektive der Betroffenen und nicht der Ausübenden von Herabsetzungen eingenommen wird, bekräftigt die Diskriminierungsforschung. Demnach neigen Pädagog_innen, die eher die Perspektive derer, die verbal diskriminieren fokussieren, dazu, dieses Verhalten als nicht böse gemeint zu bagatellisieren (Bernstein und Diddens 2021; Klocke et al. 2018). Auch wenn eine Studie zu Beweggründen und Motiven von Hatespeech darauf hindeutet, dass Herabsetzungen unter Schüler_innen soziale Funktionen wie Zugehörigkeits- und Machterleben erfüllen (Ballaschk et al. 2021), ist es wichtig, dass Pädagog_innen Hatespeech nicht ausschließlich an der Perspektive der Ausübenden festmachen, sondern Hatespeech als Reproduktion diskriminierender Machtverhältnisse und damit die Auswirkungen auf potenzielle Opfer in den Blick nehmen. Dadurch würden marginalisierte Schüler_innengruppen gestärkt und verhindert, dass sich Vorurteile und Ungleichheiten an der Schule verstärken (Fereidooni 2016). Dass Hass und Feindseligkeit von den befragten Pädagog_innen eher als Hatespeech gewertet wird, deckt sich mit Befunden aus der Gewaltforschung, wonach Lehrkräfte das Verhalten von Schüler_innen eher als gewaltvoll einordnen, je aggressiver sie es wahrnehmen (Bilz et al. 2015; Liau et al. 2004). So tendieren manche Pädagog_innen dazu, z. B. verbale Gewalt als normalen Bestandteil der Lebenswelt Jugendlicher anzusehen (Bilz et al. 2016; Yoon et al. 2004). Vor diesem Hintergrund erscheint es wichtig, dass Pädagog_innen sich bewusst sind, das Hatespeech nicht mit Hass einhergehen muss, sondern auch instrumentell eingesetzt werden kann. Zudem sollten sie frühzeitig auch bei subtilen Herabsetzungen eingreifen, da sich durch die Beleidigung sozialer Gruppen negative Einstellungen gegenüber den Adressierten verstärken können (Nicolas und Skinner 2012).

### Austragungsorte von Hatespeech aus der Sicht pädagogischen Schulpersonals

Bei der Frage, wo Hatespeech wahrgenommen wird, wurde deutlich, dass Hatespeech vorrangig als Online-Phänomen in sozialen Netzwerken angesehen wurde. Aber auch offline in der Schule wurden Hatespeech-Beobachtungen geschildert. Die Verwobenheit von Hatespeech im Online- und Offline-Kontext steht im Einklang mit Erkenntnissen der Bullying-Forschung, die ebenfalls Zusammenhänge zwischen Offline- und Cyber-Bullying aufzeigen konnte (Wachs und Wolf 2011). Für Hatespeech bei Jugendlichen sind solche Verflechtungen ein Forschungsdesiderat, da die bisherige Forschung Hatespeech entweder nur in Schulen (Lehman 2019, 2020) oder ausschließlich digital erforschte (Kansok-Dusche et al. 2022). Da Schüler_innen Hatespeech gezielt so einsetzten, dass sie sich der Wahrnehmung von Pädagog_innen entzieht, ist eine positive Beziehung zwischen Schüler_innen und pädagogischen Fachkräften förderlich, da Betroffene das Schulpersonal dann eher in Konflikte einbeziehen (Lehman 2019; Tillmann et al. 2007).

## Limitationen

Es bleibt zu reflektieren, dass den Befragten im Laufe des Interviews eine Arbeitsdefinition von Hatespeech vorgelegt wurde, welche die weiteren Erzählungen zum eigenen Verständnis von Hatespeech beeinflusst haben könnte. Zudem wurden beispielhaft soziale Gruppen benannt, die von Hatespeech betroffen sein können. Dies könnte insofern suggestive Wirkung entfaltet haben, als dass Pädagog_innen ihr Verständnis von Hatespeech auf bestimmte Gruppen fokussiert haben, während andere nicht mit betrachtet wurden. Gleichwohl haben die Pretests gezeigt, dass die Erläuterungen zur Hatespeech-Definition die Abgrenzung zu Bullying erleichterten und die exemplarische Aufzählung sozialer Gruppen den Gruppenbezug von Hatespeech besser verdeutlichen konnte. Die Studie kann zudem aufgrund des qualitativen Designs keine Aussagen dazu machen, wie häufig Schüler_innen als Angehörige sozialer Gruppen und von welchen Ausdrucksformen von Hatespeech diese online und offline betroffen sind. Weitergehende quantitative Forschung könnte hier Aufschluss geben.
